# PhotoNeuro: A compact photodetector for synchronization of visual stimulus presentation during behavioural experiments in neuroscience

**DOI:** 10.1016/j.ohx.2025.e00677

**Published:** 2025-07-12

**Authors:** Xavier Cano-Ferrer, Marcelo J. Moglie, George Konstantinou, Antonin Blot, Gaia Bianchini, Albane Imbert, Petr Znamenskiy, M. Florencia Iacaruso

**Affiliations:** a Making Science and Technology Platform, The Francis Crick Institute, 1 Midland Road, London NW1 1AT, UK; b Neuronal Circuits and Behaviour Laboratory, The Francis Crick Institute, 1 Midland Road, London NW1 1AT, UK; c Specification and Function of Neural Circuits Laboratory, The Francis Crick Institute, 1 Midland Road, London NW1 1AT, UK

**Keywords:** Photodetector, Visual stimuli, Animal behaviour, Systems neuroscience, Bonsai

## Abstract

Presenting visual stimuli in neuroscience experiments often requires precise temporal alignment between visual events and electrophysiological or behavioural recordings. This is typically achieved by combining analogue signals that convey timing information about the visual cue shown on liquid crystal displays (LCDs), sensed via photodetectors and recorded through analogue-to-digital converter (ADC) acquisition boards. However, most commercial photodetector systems pose limitations such as high voltage requirements, large sensor footprints that interfere with stimulus presentation, and limited compatibility with open-source platforms. Here, we present a compact, low-cost photodetector system designed for compatibility with common 3.3–5 V microcontroller-based development boards (e.g., Arduino) and the open-source visual programming language Bonsai, widely used in neuroscience for experiment control. The circuit consists of a photodiode, an amplification stage, and a low-pass filter, and can optionally incorporate an infrared filter—useful for experiments involving infrared touch displays. To facilitate reproducibility, we provide complete design files, a bill of materials and detailed building and operational instructions. We further introduce a four-channel variant, enabling the detection of four-bit binary signals for more complex synchronization needs. Validation and characterization of the device were performed through grayscale gamma correction analysis of LCD monitors using Bonsai. Additionally, we demonstrate the system’s utility in a head-fixed mouse experiment, synchronizing visual stimulus onset with neuronal recordings acquired via Neuropixels 2.0 probes. Performance comparisons with a commercial photodetector device indicate that our system achieves equivalent signal fidelity at a substantially lower cost, while maintaining a minimal footprint suitable for experimental use.

## Graphical abstract

**Figure:**
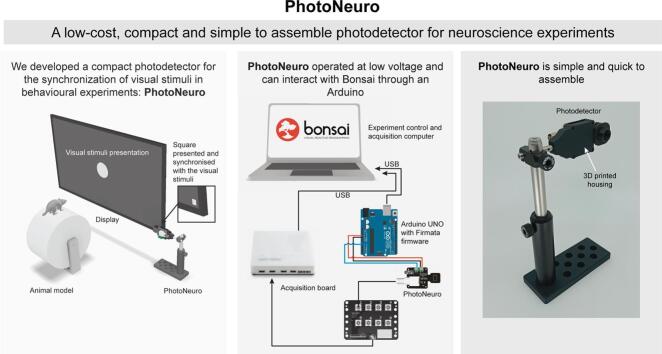

**Table:** 

Specifications table	
Hardware name	*PhotoNeuro*
Subject area	• Neuroscience
Hardware type	• Measuring physical properties and in-lab sensors
Closest commercial analog	Harp Photodiode v2.1 (https://www.cf-hw.org/harp/behavior) Sanworks Frame2TTL v3 ($395.00 USD) (https://sanworks.io/shop/viewproduct?productID=1502) Thorlabs DET36A2 (£120.62 without mounting accessories) (https://www.thorlabs.com/thorproduct.cfm?partnumber=DET36A2#ad-image-0)
Open source license	CC-By Attribution 4.0 International
Cost of hardware	Shipping costs are not included. • 67 GBP (manufacturing 1 unit) and 17 GBP (10 units) without mounting accessories. • 117 GBP (manufacturing 1 unit) and 56 GBP (10 units) with proposed mounting accessories.
Source file repository	http://doi.org/10.17605/OSF.IO/CQWD9

## 1 Hardware in context

Understanding visual processing in neuroscience requires precise control not only over the content of visual stimuli but also their timing. Accurate timestamping of stimulus onset is essential in experimental paradigms that measure reaction times in behavioural tasks or align neural recordings with stimulus events. Temporal synchronisation is especially important for quantifying neuronal response latencies, assessing the reliability of responses across repeated trials, and to determine the role of spike time precision in neural computations [1]. In cognitive and sensory neuroscience, precise timing underlies key measurements such as event-related potentials (ERPs), time–frequency responses (TFRs), and autonomic signals like pupil dilation and galvanic skin response. Even small timing inaccuracies can lead to misinterpretation of sensory or cognitive processes [2]. Moreover, in multisensory integration research, poor temporal alignment of auditory and visual cues can disrupt the perception of a unified sensory event [3].

In recent years, systems neuroscience has seen the emergence of a growing open-source ecosystem for conducting and analysing experiments. On the hardware side, OpenEphys, a multichannel electrophysiology acquisition board [4] which allows the use of silicon probes such as Neuropixels probes [5,6] and tetrode recordings [7,8]. Other experimental control tools such as Bpod [9], HARP [10] and devices from Open Source Sussex Neuroscience [11] are available for their replication. Additionally, a growing network of open-source software packages has emerged in recent years to give new tools for experimental neuroscientists such as Bonsai [12] and Bonvision [13], adding to more traditional tools such as Psychophysics Toolbox [14] and PsychoPy [15]. All these open-source hardware and software tools have created an ecosystem that enables different applications which combine behavioural arena control and automation with simultaneous stimuli presentation, electrophysiological and videography recordings in open or closed loop with low latency [16].

**Fig. 1:**
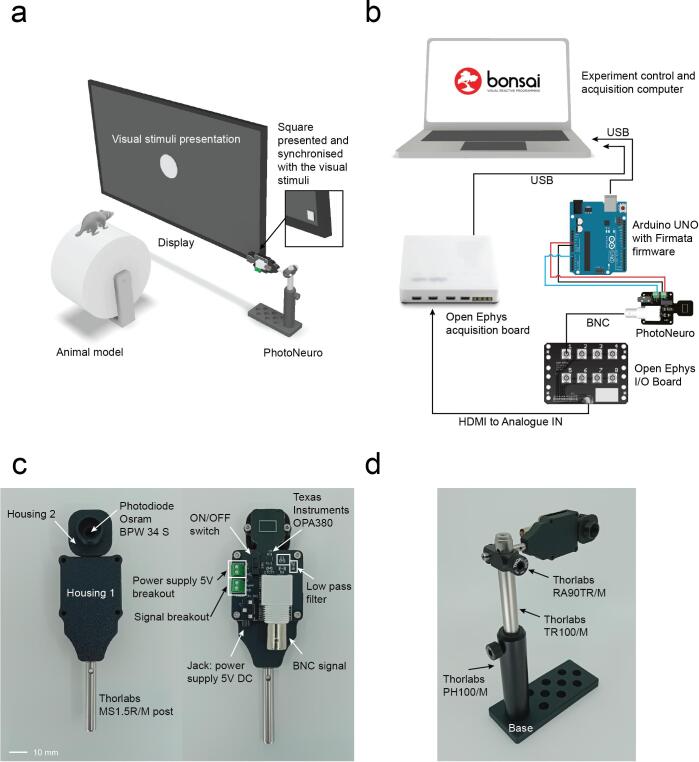
PhotoNeuro description. a. Typical application in a behavioural experiment with visual stimuli presentation. b. Electrical diagram showcasing the main photodetector application: PhotoNeuro is connected simultaneously to an acquisition board which acquires all experimental signals with high temporal resolution and an Arduino UNO controlled by a Bonsai program that controls the experiment. c. Description of the main components of the photodetector. d. Optomechanical components proposed to hold the device in place.

For precise timing of the visual stimuli presentation, direct recording of the stimulus with a photodetector is often required, as hardware timestamping solutions are not commonly available (Fig. 1a). The device presented in this paper is an open source, inexpensive photodetector that can be powered by and interfaced directly with the Arduino Microcontroller (5 to 3.3V_DC_) and that can be rapidly manufactured. In the typical setup, the photodetector is connected to the acquisition board, for deterministic analogue recording while the signal is also acquired by the Arduino development board to interact with Bonsai without the need of other power supplies (Fig. 1b). The device features a small sensing area (≈ 1.35 cm^2^) (Fig. 1c) and we propose some optomechanical components to help the researcher position the device within the experimental setup (Fig. 1d).

When using thin-film-transistor liquid–crystal display (TFT LCD) for presenting visual stimuli, pixel intensity values and display measured luminance values do not obey a linear relationship. In many cases, TFT LCD grayscale gamma correction is required previous to the experiment. This correction adjusts the digital pixel intensity values to obtain a linear increase in actual displayed pixel intensity. There are existing initiatives to perform a semi-automated red, green and blue (RGB) gamma correction using commercially available devices [17] but, they do not allow the interaction with the acquisition hardware and software we mentioned previously. Therefore, we propose a simple, user-friendly tool that can be easily replicated and seamlessly integrated into the neuroscientists’ toolbox.

## 2 Hardware description

**Fig. 2:**
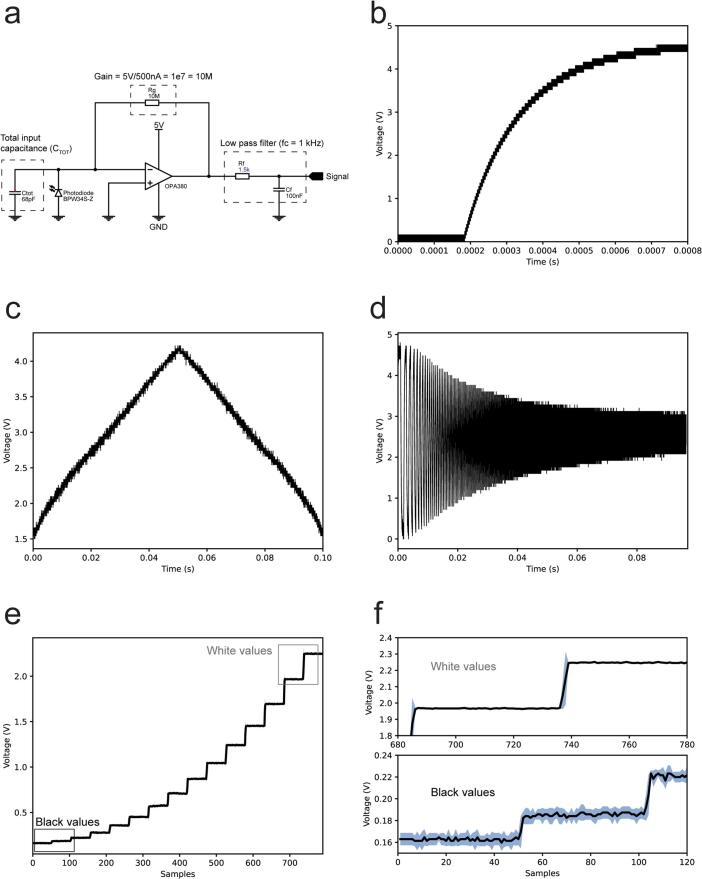
PhotoNeuro electrical characteristics. a. Main components of the analogue circuit: transimpedance amplifier, and low pass filter. b. Rise time. c. Linearity of the photodetector when measuring a light emitting diode (LED) fade in and out. d. Low pass filter response. e. Photodetector signal when a grayscale array of 16 values is presented on a Dell UP2716D display. f. Zoom in the higher and lower values of the grayscale array.

The photodetector circuit consists of a photodiode, an amplification stage and a low-pass filter. The design of the amplification stage is based on the OPA380 transimpedance amplifier (Texas Instruments Inc.). This choice was mainly motivated by its availability in single supply rail 2.7–5.5 V, high precision, long term stability and low noise with 25 μV (max) offset voltage. The photodiode selected, the BPW 34 S (ams-OSRAM AG.) offers a wide spectral range of sensitivity (400 nm to 1100) and a short switching time (20 ns). The system features a simple first order resistor–capacitor (RC) filter with a cutoff frequency (f_c_) of 1 kHz. Selecting a f_c_ of 1 kHz ensures compatibility with most ADCs used in neuroscience and it is still above most commercial display frame rates. The analogue circuitry has been designed following the transimpedance amplifier design guide on the datasheet (page 11). The following components of the circuit (Fig. 2a) were calculated as follows:

The total input capacitance $C_{\mathrm{TOT}}$ includes the parasitic common-mode $C_{\mathrm{CM}}$ and differential-mode $C_{\mathrm{DM}}$ input.

capacitance 3 pF + 1.1 pF for the OPA380 and 72 pF of the Photodiode capacitance $C_{P}$ as shown in Eq. (1):

(1) $$C_{\mathrm{TOT}}=C_{\mathrm{CM}}+C_{\mathrm{DM}}+C_{P}=3\;pF\;+\;1.1\;pF+\;72\;pF≅\;76\;pF$$

The nearest commonly available value of 68 pF was chosen.

The transimpedance gain $R_{f}$ with the photodiode current $I_{P}$ has been calculated using Eq. (2):

(2) $$R_{f}\;=\frac{V_{O}}{I_{P}}=\frac{5\;V}{500\;nA}\;=\;10\;M\Omega$$

The filter values for the capacitor C and the resistor R are extracted from Eq. (3) using a f_c_ of 1000 Hz:

(3) $$\mathrm{RC}\;=\frac{1}{2\pi f_{c}}=\frac{1}{2\pi \cdot 1000\;Hz}≅1.6{\cdot 10}^{-4}$$

R=1.5kΩ

C=100nF

The rise time measured after the filter is approximately 330 microseconds. The rise time signal was generated by a 622 nm light emitting diode (LED) (Vishay TLCR5800) connected to a function generator (RS Components RSDG 2082X) and acquired using a Siglent SDS1104X-U oscilloscope (Fig. 2b).

To assess the linearity of our hardware, a linear ramp of increasing luminance was generated by the same 622 nm LED connected to a function generator (RS Components RSDG 2082X) and acquired using a Tektronix MDO3104 oscilloscope. The signal generated was a pulse width modulated (PWM) signal at 60 kHz doing a sweep of the duty cycle from 0 to 100 % in a period of 100 ms. Results showed the linear behaviour desired. The same behaviour can be found on the photodiode datasheet in the photocurrent/open-circuit voltage relationship displayed in page 5 (Fig. 2c).

The filter response signal was generated by the same 622 nm LED connected to a function generator (RS Components RSDG 2082X) and acquired using a Tektronix MDO34 oscilloscope. The signal generated was a square wave frequency sweep with a duration of 0.1 s and a range of 0 to 10 kHz (Fig. 2d).

Ultimately the photodiode grayscale sensitivity was evaluated presenting a ramp of gray values ranging from black to white without any gamma correction (Fig. 2e). This experiment was repeated three times in order to get the standard deviation. We have verified the average standard deviation (∼0.007 V) is significantly smaller than the smallest voltage increment corresponding to the lower values of black displayed on the screen (∼0.02 V) (Fig. 2f).

The summary of the key features for the design we propose:

• Compatible with Arduino boards (it can be powered by the 5 V pin of the Arduino board and the analogue signal range is 0 to 5 V).
• Low cost (17––67 GBP for the essential materials of 1 and 10 units respectively) and easy to replicate.
• Low power consumption 37.5 mW (7.5 mA at 5 V).
• It can be recorded using commonly available ADC boards (e.g., Open Ephys, National Instruments acquisition boards)
• It can be used as a trigger signal to take decisions on the behaviour (feedback to Arduino board) Applications in Bonsai.
• Sensitivity to detect at least 16 grey levels so it can be used to record different stimuli/contrasts/colours displayed on the screen. It can be used for the grayscale gamma correction of LCD displays.
• It occupies minimal real estate area on the screen and maximises available stimuli presentation surface area.
• Additionally, it has the option to add IR filter for use with infrared touchscreens.

## 3 Design files summary

**Table:** 

Design file name	File type	Open source license	Location of the file
Schematic	PDF	CC-By Attribution 4.0 International	OSF
Printed circuit board	Easy EDA/ Gerber/csv	CC-By Attribution 4.0 International	OSF
Housing 1	Autodesk Inventor/STL/Step	CC-By Attribution 4.0 International	OSF
Housing 2	Autodesk Inventor/STL/Step	CC-By Attribution 4.0 International	OSF
Base	Autodesk Inventor/STL/Step	CC-By Attribution 4.0 International	OSF
GammaCalibration_FitGray	Bonsai	CC-By Attribution 4.0 International	OSF

*GammaCalibration_FitGray.bonsai* is a Bonsai program that generates an array of grayscale values and presents them in a square located on the bottom right corner of the display. Simultaneously, it reads the signal from the analogue pin connected to the photodetector signal and automatically applies a gamma correction. The photodetector needs to be located in front of the square.

## 4 Bill of materials summary

**Table:** 

Designator	Component	Number	Cost per unit −currency	Total cost − currency	Source of materials	Material type
PCB	Printed circuit board with assembled components	1	GBP 4.10	GBP 4.10	JLCPCB	Semiconductor Composite
Housing 1	3D printed body	1	GBP 0.26 (40 % Infill PLA)	GBP 0.26 (40 % Infill PLA)	Bambu Lab	Polymer
Housing 2	3D printed photodetector housing	1	GBP 0.06 (40 % Infill PLA)	GBP 0.06 (40 % Infill PLA)		Polymer
Base	Base to hold the optomechanical components	1	GBP 0.72 (40 % Infill PLA)	GBP 0.72 (40 % Infill PLA)		Polymer
BPW 34 S	Photodiode	1	GBP 0.66	GBP 0.66	Mouser	Semiconductor
OPA380	Precision, High-Speed Transimpedance Amplifier	1	GBP 5.82	GBP 5.82	RS Components	Semiconductor
M2 Threaded inserts	Brass threaded insert	4	GBP 15.49	GBP 15.49	Amazon	Metal
M3 Threaded inserts	Brass threaded insert	1				
M2 x 8 mm screws	Screws to hold housing 2	2	GBP 0.65	GBP 1.3	Accu	Metal
M2 x 4 mm screws	Screws to hold the PCB	4	GBP 0.65	GBP 2.6	Accu	Metal
M6 x 12 mm screws	Screws to attach the optical post holder with the base	3	GBP 0.7	GBP 2.1	Accu	Metal
Mono jack cable	Power supply cable	1	GBP 1.05	GBP 1.05	Amazon	Other
BNC cable	BNC to BNC Cable − 1 m	1	GBP 3	GBP 3	PiHut	Other
Thorlabs MS1.5R/M	Optical post: 6 mm diameter and 38 mm length	1	GBP 6.13	GBP 6.13	Thorlabs	Metal
Thorlabs RA90TR/M	Right angle optical post clamp	1	GBP 14.54	GBP 14.54	Thorlabs	Metal
Thorlabs TR100/M	Optical post: 12.7 mm diameter and 100 mm length	1	GBP 5.34	GBP 5.34	Thorlabs	Metal
Thorlabs PH100/M	Optical post holder: 100 mm length	1	GBP 8.54	GBP 8.54	Thorlabs	Metal
Infrared filter (optional)	Infrared filter required for infrared touchscreen displays	1	GBP 0.969	GBP 0.969	Amazon	Other

## 5 Build instructions

### 5.1 Required tools

Vice
Soldering station
Flux
1.5 mm hex key
2 mm hex key
5 mm hex key

### 5.2 Ordering the printed circuit board

The Printed circuit board (PCB) manufacturing files are available for download in the PhotoNeuro OSF repository. Three files are required for manufacturing the device electronics: the Gerber file which contains the settings for the PCB, the pick and place file that contains the coordinates to place the electronic components and the bill of materials (BOM) that has the reference of all components. For the manufacturing of the device, we used JLCPCB because the BOM file contains all their references and the device has been designed based on around their component stock therefore, only two components needed to be soldered manually. The steps for ordering the board are shown in a manufacturer video.

1. Download the PhotoNeuro OSF repository files.
2. Create customer account on JLCPCB and sign in.
3. Upload the Gerber file on the instant quote.
4. Select preferred PCB colour, quantity and leave default settings.
5. On the bottom of the page, enable SMT assembly and indicate how many PCB need to be assembled from the total PCB ordered.
6. Click next and on the next page add the BOM and pick and place files.
7. Click next and review if any parts are not available. The stock can change and some parts may require ordering from other suppliers (e.g. Farnell, DigiKey, RS Components).
8. Click next, save to cart and complete payment.

### 5.3 Device required components

**Fig. 3:**
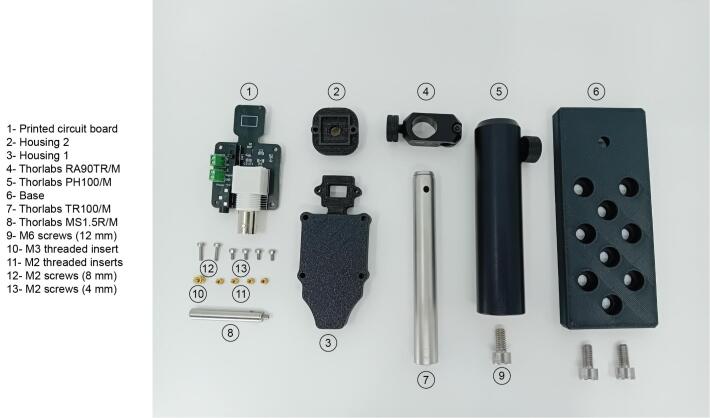
Summary of all components required for the PhotoNeuro assembly.

The components required to replicate the device are summarised in Fig. 3 and they include the printed circuit board, the 3D printed components and some optomechanical components.

### 5.4 Manufacturing of 3D printed parts

**Fig. 4:**
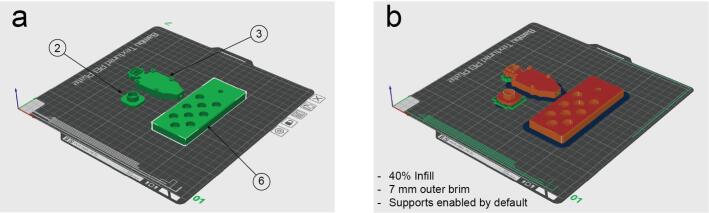
Configuration for the 3D printing process. a. Orientation of the parts. b. Settings used on a Bambu labs X1 Carbon 3D printer and appearance after slicing process.

Parts number 2, 3 and 6 have been printed using PLA Matte black filament in a Bambu Lab X1-Carbon 3D Printer using 40 % infill, supports enabled and 7 mm outer brim (Fig. 4).

### 5.5 Soldering the transimpedance amplifier and the photodiode

**Fig. 5:**
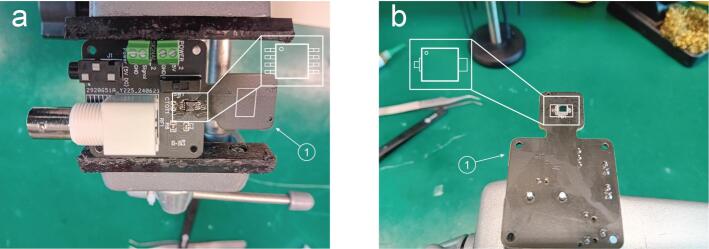
Soldering of the main two components. a. Orientation of the OPA380 transimpedance amplifier package. b. Orientation of the BPW 34 S photodiode package.

The operational amplifier and the photodiode are not available for assembly by the manufacturer. We propose these two components to be soldered manually. In order to solder the OPA380 on the PCB (Part 1) has to be clamped on the vice with the BNC connector facing upwards and use of lead-free solder flux is recommended to improve the solder distribution across the legs of the integrated circuit (IC). The orientation of the IC (top left corner dot engraved on the package) must be coincident with the silkscreen (Fig. 5a). The next step starts by flipping the PCB, hold it in the vice and solder the photodiode with the dot oriented on the left top corner (Fig. 5b).

### 5.6 Threaded inserts

**Fig. 6:**
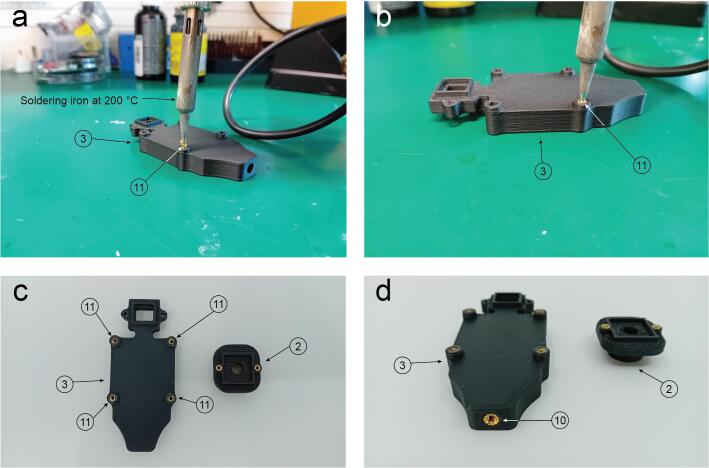
Threaded inserts assembly. a. A soldering iron at 200 °C is compressing the part 11 (M2 threaded insert) into the hole. b. The metallic insert is flush with the plastic surface. c. The step is repeated for parts 2 and 3 (Housing 1 and 2). d. Detail of the M3 threaded insert placed on part 3 (Housing 1).

Use the soldering iron at temperature of 200 °C to insert the M2 brass threaded inserts (Part 11) (Fig. 6a and b). The step is repeated for parts 2 and 3 (Fig. 6c). Finally, an M3 insert is also placed into the body of part 3 (Fig. 6d).

### 5.7 Assembly of the photodetector

**Fig. 7:**
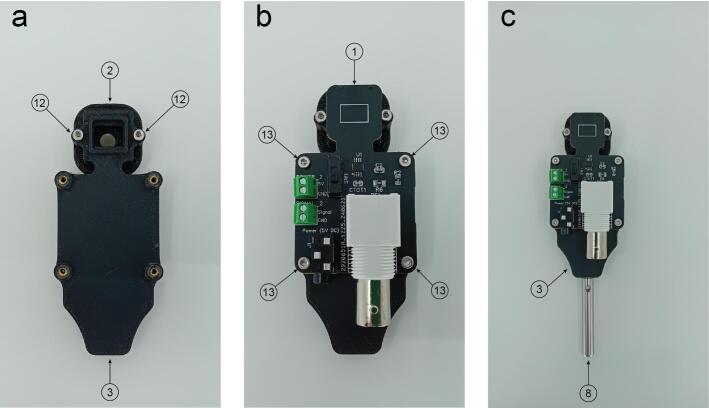
Photodetector main body assembly. a. Parts 2 and 3 are joined together using two M2 screws (Part 12). b. The printed circuit board (Part 1) attached with parts 2 and 3 using four M2 screws (Part 13). c. Part 8 connects to Part 3 through an M3 grub screw.

Parts 2 and 3 are attached together using two M2 screws (Part 12) (Fig. 7a). Then the PCB (Part 1) can be attached with the previous construct by using four M2 screws (Part 13) (Fig. 7b). As a final step screw the part 8 on the bottom of part 3 (M3 thread) (Fig. 7c).

### 5.8 Assembly of the photodetector with the rest of optomechanical components

**Fig. 8:**
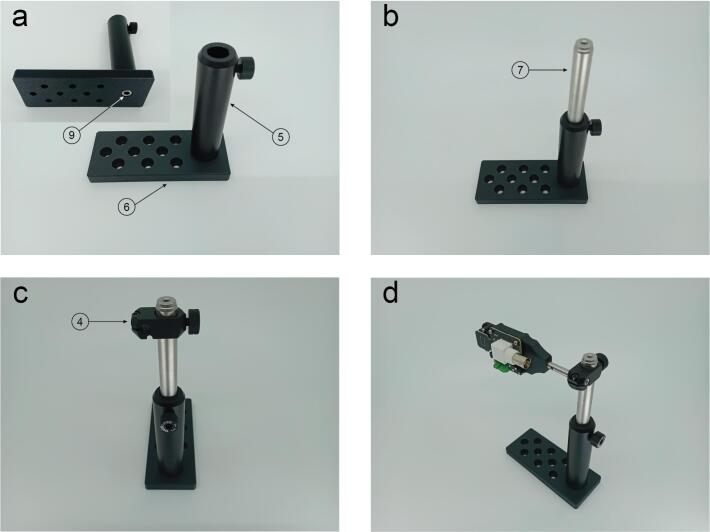
Optomechanical components assembly. a. The base (Part 6) is connected to Thorlabs PH100M (Part 5) using a M6 screw (Part 9). b. The part 7 (Thorlabs 12.7 mm post) slides inside and it is held in position with the thumb screw of part 5. c. Thorlabs RA90TR/M (Part 4) slides surrounding the 12.7 mm post. d. The photodetector body assembled in previous Fig. 7 is assembled with the optomechanical components.

Parts 5 and 6 are attached together using an M6 screw (Fig. 8a). Then slide part 7 inside part 6 and tighten it using the thumb screw on part 6 (Fig. 8b). Part 4 slides on part 7 and it is also adjusted using its thumb screw (Fig. 8c). Then slide the photodetector assembled in the previous step (Fig. 7c) and hold in in place by tightening the screw using a 2 mm hex key (Fig. 8d).

## 6 Operation instructions

The main components of the photodetector (transimpedance amplifier and photodiode) are electrostatic-sensitive devices and therefore the operator is advised to operate the device in an ESD-safe working environment such a grounding mat.

In order to operate the device, the 5 V and the GND must be connected to the power supply, we recommend using the 5 V and GND pins of an Arduino board or similar either connecting them to the screw terminals or the Jack connector.

Then the signal pin can be connected to the Arduino board using the screw terminal and the BNC can be connected to the acquisition board. The GND pin should also be connected to the acquisition board.

The device output voltage signal range varies from 0 V to 5 V, if you are using a development board that is not 5 V logic voltage or 5 V tolerant you could permanently damage the analogue to digital converter (ADC) peripheral.

The photodetector is a low power device (37.5 mW and 7.5A) and does not present potential safety hazards for users but, electrical work (e.g., wiring, soldering, etc.) must be conducted under supervision of a licensed electrician or equivalent experience/knowledge.

## 7 Validation and characterization

### 7.1 Grayscale gamma correction of LCD displays using Bonsai

**Fig. 9:**
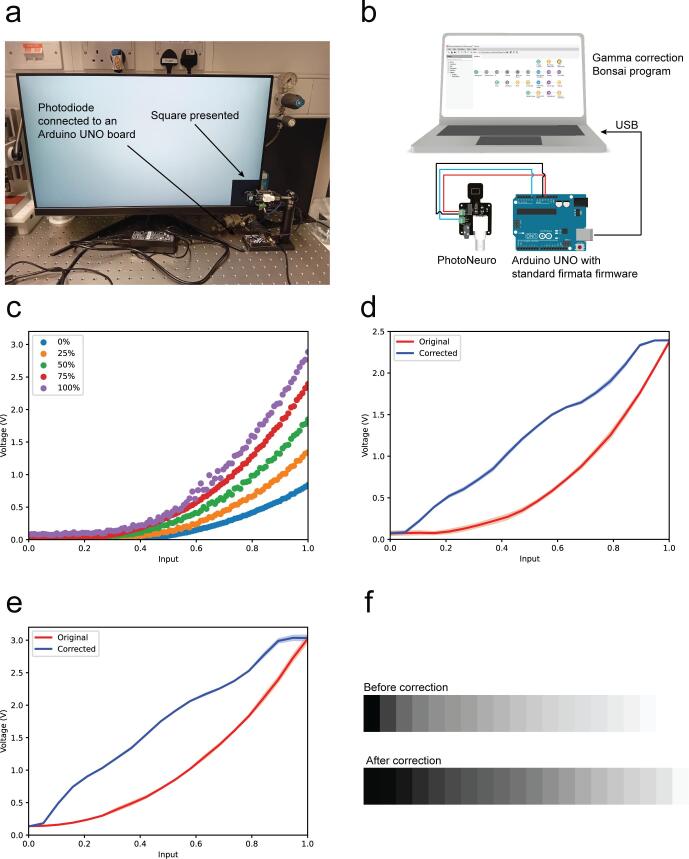
Use of the photodetector to perform a liquid–crystal display (LCD) gamma calibration. a. The experimental setup where PhotoNeuro is placed against the display and an array of 100 values of grey ranging from black to white are presented in a square on the right bottom side of the display. b. Electrical connections required to replicate the experiment. c. Grayscale ramp of 100 values spaced 200 ms at five different brightness levels on the msi G2422 display. d. Original and corrected curves for msi G2422 display. e. Original and corrected curves for Dell UP2716D. Three replicates were acquired (error bars show ± standard deviation). f. Gamma LUT of msi G2422 display before and after the calibration.

Monitors display light in a nonlinear manner, typically with a gamma value of approximately 2.2 [17]. To linearly control luminance for neuroscience and behavioural applications, a grayscale gamma calibration is required. The first application we present on this manuscript is the gamma correction of TFT LCD. The setup requires the photodiode to be located against the display where the stimuli will be presented, in our case the bottom right corner and the calibration should be carried out in darkness (Fig. 9a). The second step is wiring the PhotoNeuro to an Arduino UNO® and connecting the system to the computer running the Bonsai program *GammaCalibration_FitGray.bonsai* (Available on the repository in the Bonsai folder), via USB. The program displays a grayscale ramp of 20 values that are displayed at one intensity level per second (Fig. 9b). The reason behind choosing 20 samples displayed in 20 s was based on what is proposed by the BonVision gamma calibration tutorial [18] and no significant difference was observed when acquiring more samples. We first acquired the photodetector signal with different brightness values (0–100 %) set up on the display. In that case, the number of gray steps was 100, with each one displayed for 200 ms (Fig. 9c). After setting the display intensity level to 75 %, the gamma calibration routine was performed for two different TFT LCD displays: a Dell UP2716D and an msi G2422, three times each. The results showed high repeatability with both displays (Fig. 9d-e). The Bonsai code saves the data on a CSV file and generates a gamma look up table (gammaLUT) bitmap for its further use [17] (Fig. 9f).

### 7.2 Comparison with a commercially available device

**Fig. 10:**
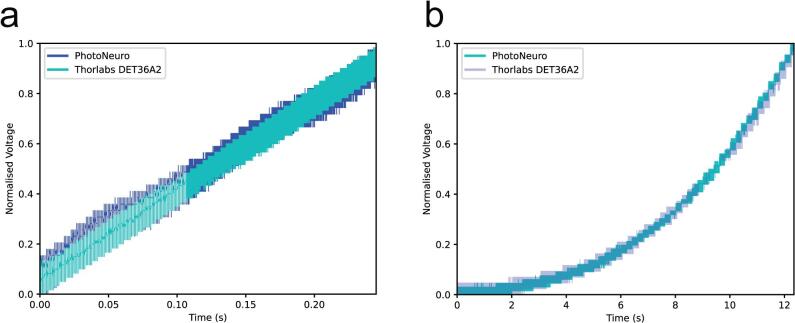
Comparison with commercial option. a. PhotoNeuro and Thorlabs DET36A2 on a PWM signal emitted by an LED b. PhotoNeuro and Thorlabs DET36A2 voltage signals while displaying a grayscale ramp of 64 values spaced 200 ms on the msi G2422 display. Values of voltage have been normalised.

We have compared the photodetector with a commercially available option: the Thorlabs DET36A2 biased photodetector which operates at 12V_DC_ and has a higher signal amplitude. We firstly acquired both signals simultaneously using a Siglent SDS1104X-U oscilloscope. The luminous signal was generated by the LED used in previous experiments, driven by a function generator (RS Components RSDG 2082X). The signal was a 100KHz PWM pulse train (0 to 5 V) with its duty cycle linearly modulated (0 to 100 %) over 0.24 s (Fig. 10a). The signal output of both photodetectors when measuring a grayscale ramp of 64 values spaced 200 ms is consistent with the previous linearity results obtained (Fig. 10b). This experiment was performed to confirm the electrical characteristics and linearity observed in Fig. 2.

### 7.3 Optional infrared filter

**Fig. 11:**
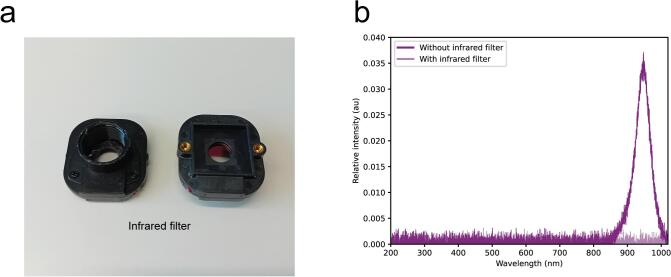
Infrared filter. a. Physical appearance of the infrared filter. b. Signals corresponding to a 940 nm LED light source with and without the filter in place, the data was recorded using a Thorlabs compact spectrometer (CCS200/M).

As optional feature, the 3D printed part Housing 2 can be replaced by an infrared filter provided in the bill of materials. That is useful in applications with infrared touch displays where an array of infrared emitters-receivers is surrounding the display, therefore the signal from touch detection registered by the photodetector would have a higher magnitude than the display signal (Fig. 11a-b). This option has been successfully tested with the infrared touch screen NIB 230 (Nexio Co., Ltd.).

### 7.4 Four channel option

**Fig. 12:**
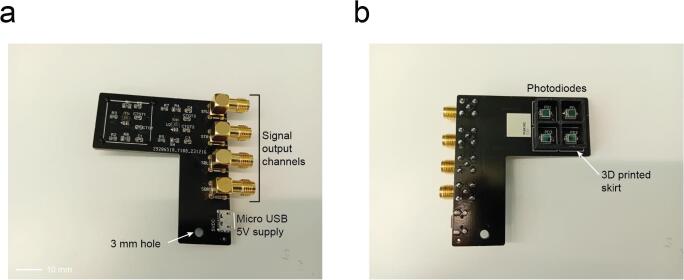
Four-channel version of the photodetector. a. Top layer components description. b. Bottom layer components description.

Additionally, for researchers who need to record four bits of binary signals, we have designed and manufactured a four-channel version which has two OPA2380 (dual channel version of the OPA380) resulting on four individual channels. The four photodiodes (BPW 34 S) are confined in a small area (20 x 20 mm) and the four amplified analogue signals are connected to four SMA connectors. The board has a 3 mm hole to connect to a MS1.5R/M rod or similar and hold it in place (Fig. 12a and b).

### 7.5 In vivo validation

**Fig. 13:**
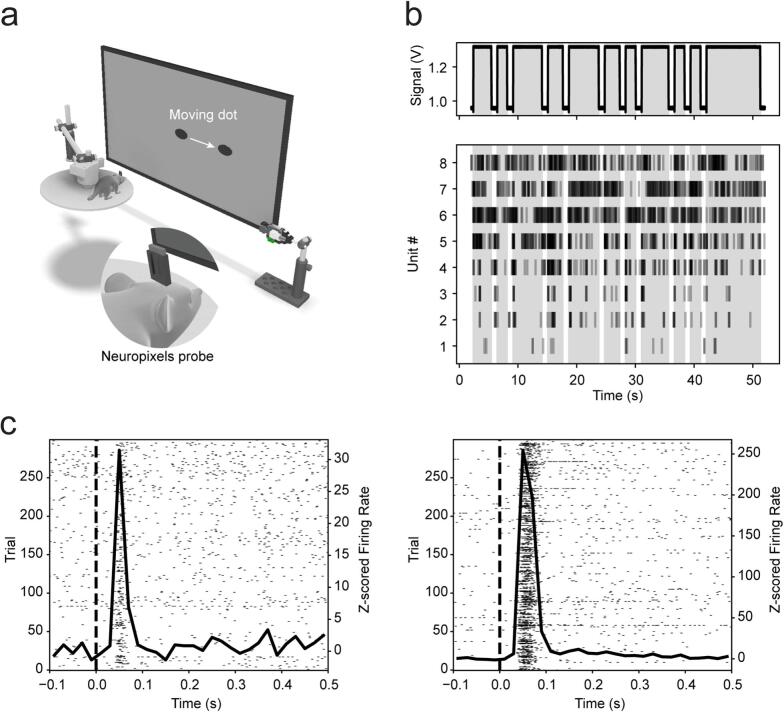
In vivo validation of PhotoNeuro. a. Schematic of experimental setup. b. Top: Voltage signal obtained from PhotoNeuro. Gray-shaded areas indicate stimulus presentation, white shaded areas indicate inter stimulus interval. Bottom: Example responses from 8 units recorded from the superior colliculus of head-fixed mice aligned to the PhotoNeuro signals above. C. Response profiles for two example neurons. The spike raster plots (spiking activity for 300 repetitions) are overlaid with the peristimulus time histogram (PSTH) during visual stimulation.

To evaluate the functionality of PhotoNeuro, we employed it to synchronize neuronal responses with visual stimuli presented on an LCD monitor Nexio NIB 230 (Nexio Co., Ltd) with 508.15(H) × 285.7(V) mm display area at 60 Hz. Experiments were conducted in head-fixed C57BL/6Jax mice implanted with chronically mounted Neuropixels 2.0 probes [19] (Fig. 13a). Electrophysiological recordings were performed using a National Instruments PXIe-6341 I/O module and the Open Ephys software platform [4]. The monitor was positioned 32 cm in front of the animal, which was placed on a running wheel. Visual stimuli consisted of black ellipses (dimensions: 3 cm width – 1.5 cm height) moving at various speeds (0, 5, 10, 15 cm/s), randomly interleaved, and presented on a gray background. Each stimulus was shown for a variable duration (0.8–10 s), with an inter-trial interval of 1 s. At stimulus onset and continuing until the ellipse exited the screen, a white square appeared in the bottom right corner of the display. This visual marker was detected by PhotoNeuro, enabling precise synchronization with the neuronal recordings from the superior colliculus, acquired using the National Instruments board (Fig. 13b).

To process the recorded neuronal responses, sessions were first automatically spike-sorted using Kilosort2 [20], followed by manual curation in Phy [21] to isolate single units. Units were classified as ‘good’ based on the following criteria: presence of a strong refractory period in the cross-correlogram, a typical waveform shape, and a stable firing rate across the session. Following synchronization to stimulus onset and averaging across trials, we observed clear visually evoked responses in neurons located in the superficial visual layers of the superior colliculus, as demonstrated by the example units shown in Fig. 13c. The Bonsai code to generate the visual stimulation protocol, pre-processed data and code to generate Fig. 13 are available in the repository (Neuropixels data folder).

## Ethics statement

Mice were bred and maintained at the mouse facility of the Francis Crick Institute, with controlled temperature (21 ± 2 °C) and humidity (55 ± 10 % RH), with restricted access to a food diet (Teklad global diet, Envigo, UK) and *ad libitum* access water, and kept on a 12  h:12  h light/dark cycle (lights on at 10 pm). All procedures were conducted in accordance to the United Kingdom Animal (Scientific Procedures) Act 1986, approved by institutional Animal Welfare and Ethical Review Body (The Francis Crick Institute PPL Review Committee) and conducted under authority of the Home Office approved Project Licence PP2817210.

## CRediT authorship contribution statement

**Xavier Cano-Ferrer:** Writing – review & editing, Writing – original draft, Visualization, Validation, Methodology, Formal analysis, Data curation, Conceptualization. **Marcelo J. Moglie:** Writing – review & editing, Validation, Software, Methodology, Data curation, Conceptualization. **George Konstantinou:** Writing – review & editing, Validation. **Antonin Blot:** Writing – review & editing, Data curation, Conceptualization. **Gaia Bianchini:** Writing – review & editing, Data curation. **Albane Imbert:** Writing – review & editing, Conceptualization. **Petr Znamenskiy:** Writing – review & editing, Conceptualization. **M. Florencia Iacaruso:** Writing – review & editing, Conceptualization.

## Declaration of competing interest

The authors declare that they have no known competing financial interests or personal relationships that could have appeared to influence the work reported in this paper.
